# Effects of inulin supplementation on body composition and metabolic outcomes in children with obesity

**DOI:** 10.1038/s41598-022-17220-0

**Published:** 2022-07-29

**Authors:** Chonnikant Visuthranukul, Supakarn Chamni, Tanisa Kwanbunbumpen, Puthita Saengpanit, Yuda Chongpison, Surapun Tepaamorndech, Ekkarit Panichsillaphakit, Jaraspong Uaariyapanichkul, Natthapong Nonpat, Sirinuch Chomtho

**Affiliations:** 1 Pediatric Nutrition Research Unit, Division of Nutrition, Department of Pediatrics, Faculty of Medicine, Chulalongkorn University, 1873 Rama 4 Road, Pathumwan, Bangkok, 10330 Thailand; 2 Natural Products and Nanoparticles Research Unit, Department of Pharmacognosy and Pharmaceutical Botany, Faculty of Pharmaceutical Sciences, Chulalongkorn University, Bangkok, 10330 Thailand; 3 Division of Nutrition, Department of Pediatrics, King Chulalongkorn Memorial Hospital, The Thai Red Cross Society, Bangkok, 10330 Thailand; 4 The Skin and Allergy Research Unit, Faculty of Medicine, Chulalongkorn University, Bangkok, 10330 Thailand; 5 Biostatistics Excellence Center, Research Affairs, Faculty of Medicine, Chulalongkorn University, Bangkok, 10330 Thailand; 6 National Center for Genetic Engineering and Biotechnology (BIOTEC), Pathumthani, 10210 Thailand; 7 Department of Microbiology, Faculty of Medicine, Chulalongkorn University, Bangkok, 10330 Thailand

**Keywords:** Paediatric research, Clinical trial design

## Abstract

Inulin might improve body composition in obese children. We aimed to determine the effects of inulin supplementation on body composition and metabolic outcomes in obese children. A randomized, double-blinded placebo-controlled study was conducted in obese Thai children aged 7–15 years. Participants were assigned to 3 treatment groups for 6 months: 13 g of extracted inulin powder from Thai Jerusalem artichoke, isocaloric maltodextrin, and dietary fiber advice groups. Body composition was assessed by bioelectrical impedance analysis. One-hundred and fifty-five children completed the study (mean age 10.4 ± 2.2 years, BMI z-score 3.2 ± 1.0, 59% male). The drop-out rate was 6%. The inulin extract yielded more than 90% compliance without significant gastrointestinal side effects. All three groups demonstrated a significant decrease in BMI z-score, fat mass index (FMI), and trunk FMI, but the differences between groups were not observed. Fat-free mass index significantly increased only in the inulin group (16.18 ± 1.90 vs. 16.38 ± 1.98 kg/m^2^, P = 0.009). There were no significant differences in the metabolic profiles between groups. Despite showing no substantial effect on adiposity, inulin may increase fat-free mass in obese children. Further research in the change of gut microbiota composition is needed to determine inulin’s impact on host-microbe interaction in pediatric obesity.

## Introduction

Obesity is a major concern worldwide. The prevalence of overweight and obesity among children and adolescents has risen dramatically from 4% in 1975 to over 18% in 2016^1^. The overweight and obese children are at risk of developing co-morbidities such as type 2 diabetes mellitus, hypertension, dyslipidemia, metabolic syndrome, non-alcohol fatty liver disease, and premature cardiovascular diseases^2,3^. Furthermore, obese children are highly prone to become obese adults, especially when having a high body mass index (BMI) or an obese parent^4^.

At present, the most important strategies to manage childhood obesity are therapeutic lifestyle changes. However, these are often difficult to achieve. Additionally, emerging evidence suggests that the bacterial dysbiosis within the gut may involve in the pathogenesis of obesity^5^. Several studies have shown differences in gut microbiota composition between obese and lean subjects^5–8^. Thus, consumption of prebiotics, which are non-digestible food ingredients utilized by gut microorganisms and beneficially affect host physiology, could be one such strategy to manage obesity in children and adults^8^. Oligofructose, an inulin-type fructan (ITF), is known to have prebiotic properties^9^. It has been shown that oligofructose fermentation normalized gut microbiota dysbiosis, leading to the modulation of gastrointestinal hormones that promote satiety, and decrease inflammation^10^. A systematic review and meta-analysis revealed significant reductions in BMI, body weight, and body fat after soluble fiber supplementation in overweight and obese adults^11^. However, the studies on body weight and body fat in overweight and obese children are very scarce and show inconclusive outcomes. Liber et al. studied the effect of oligofructose supplementation on BMI in overweight and obese children. The BMI-for-age z-score difference and total body fat did not differ between the control and experimental groups^12^. On the other hand, another study in overweight or obese children determined the positive effects of prebiotics on body weight and body composition^13^. These indicate that studies of prebiotic effects on body composition and metabolic outcomes in obese children are still inconclusive and may differ between adult and pediatric populations. Additionally, a difference in ethnicity and the amount of usual dietary fiber intake may contribute to different health outcomes. Thus, we aimed to determine the effects of prebiotic (as inulin) supplementation on body weight, adiposity, and metabolic profiles in obese Thai children.

## Methods

### Subjects

This was a randomized double-blinded placebo-controlled trial conducted from August 2017 to July 2020 at the King Chulalongkorn Memorial Hospital (KCMH), Thailand. The study was conducted in accordance with the Declaration of Helsinki and the Ethics Committee of the Faculty of Medicine, Chulalongkorn University approved the study protocol (IRB no. 240/60). The participants signed informed consent forms prior to enrollment. This trial was registered at http://www.clinicaltrials.gov as NCT03968003. CONSORT was done as the health research reporting checklist for randomized trial. Obese children aged between 7 and 15 years who had BMI above median plus 2 standard deviations (SDs) from the WHO growth reference^14^ were recruited from the Pediatric Nutrition and the Pediatric Obesity clinics from the KCMH, as well as from the social media (Chula Kids Club). All the children who met the inclusion criteria together with their parents were approached by the researchers and those agreed to the monthly follow-up and consented to the study were enrolled. The exclusion criteria were syndromic obesity, endocrine causes of obesity (e.g. hypothyroidism, growth hormone deficiency), concomitant use of medications that influence appetite or body weight (e.g. corticosteroids), and attending other concurrent weight reduction programs.

### Study design

Participants were randomly allocated to 3 groups: inulin, placebo, and dietary fiber advice group. Randomization was performed by means of computer-generated permuted blocks size of 6. A research assistant who did not participate in data collection and analysis, generated the random allocation sequence and prepared the sealed envelopes. Other researchers enrolled participants and blindly assigned them to each group. To ensure concealment, the study products were weighed, packaged, and signed by consecutive numbers according to the randomization list by non-involved personnel. The inulin group consumed 13 g of extracted inulin powder from Thai Jerusalem artichoke around 30 min prior to dinner daily. The participants were recommended to consume the powder by mixing the powder to 150 ml of warm water and then stirring up the powder until dissolved. The placebo group consumed 11 g of isocaloric maltodextrin (Oligocarb; Ma-Jusmin Company Limited, Bangkok, Thailand) in the same manner. Blinding of the placebo to match the light brown color of inulin extract were processed through the study. Both supplements were provided in identical foil sachets. All participants were followed-up monthly for 6 months. During the monthly visit, the participants were asked to return all empty, half-empty, and full sachets, and the information was recorded. The parents/guardians were asked to do a parental checklist and verified with the researchers during the follow-up visits. The third group received structured advice (with portion size pictures) to consume appropriate amounts of dietary fiber for age^15,16^. Research assistants performed telephone contacts every 1st and 3rd week to check and monitor compliance and side effects.

Apart from the interventions, all groups received the same dietary advice about low energy (1,000–1,200 kcal/day) and low fat (25% of total energy from fat) diet. All participants were instructed to exert non-weight-bearing exercise for 60 min per day at least 4–5 days per week, to maintain a physical active lifestyle, and to reduce screen time.

### Inulin extraction process

Inulin was prepared from Jerusalem artichoke (*Helianthus tuberosus*). Dry power of Jerusalem artichoke was purchased from local distributors in Nakhon Ratchasima, Thailand. The product was approved for human consumption by the Food and Drug Administration of Thailand (No. 30–1-14,358–1-001). The inulin extract was prepared based on the patented protocol developed by our team (Patent no. 15858, Inventor: Chonnikant Visuthranukul and Supakarn Chamni, Chulalongkorn University and National Science and Technology Development Agency, Thailand). Briefly, Jerusalem artichoke powder was decocted in water at 60-80ºC for 45–90 min. The resulting slurry was filtered, mixed with absolute ethanol, and incubated at room temperature overnight for inulin precipitation. Inulin was collected by filtration. All remained moisture contents were dried at 40-50ºC and pulverized to obtain the fine and homogenous inulin powder. The inulin content was evaluated based on the standard methods for dietary fiber including energy (ASTM Method D 240–76), total dietary fiber (AOAC (2016) 985.29), soluble dietary fiber (AOAC (2016) 991.42, 991.43), insoluble dietary fiber (AOAC (2016) 991.42), and fructans (Inulin + Oligofructose) (s) AOAC (2005) 997.08 + J. AOAC, 2000, 83(4); 1020–1025)^17,18^. The tests were performed by certified laboratory of the Institute of Nutrition, Mahidol University, Thailand. The degree of polymerization (DP) of resulting inulin was determined by MALDI-TOF Mass Spectrometry and Gel Permeation Chromatography coupled to multiangle laser light scattering. Jerusalem artichoke inulin indicating great distribution of fructan polymerization containing molecular mass 3460 m/z, which referred to the large DP of the resulting inulin extract. The microbial and heavy metal contaminants, and pesticide residues were examined and confirmed by the Department of Science Service, Thailand. The inulin extract was kept in aluminum foil sachets and stored in a dry container at ambient temperature until use.

### Assessment of dietary intake, physical activity, and exercise

Dietary intake was assessed by a dietician, using 3-day dietary records (two weekdays and one weekend day). Fiber and other nutrient intakes were calculated using the Institute of Nutrition, Mahidol University Calculation-Nutrients (INMUCALs) Version 3^19^. Adherence to the instruction of physical activity and exercise was evaluated by a physical activity questionnaire at every visit. The intensity of physical activity was defined by day/week and duration of physical activity in minutes/hours. Aerobic dance or bicycle riding with speed was classified as high intensity exercise. Brisk walking was classified as moderate intensity whereas walking from one place to another for at least 10 min was categorized as low intensity. Sedentary activity was defined as a type of lifestyle involving little or no physical activity which was interviewed and assessed by a research assistant. Screen time, such as watching television and playing games on the computer, smart phone, and tablet, was assessed separately from sedentary activity.

### Anthropometry, body composition, and clinical evaluation

Trained personnel performed the anthropometric measurement. Weight and height were measured without shoes and with light clothing using a stadiometer to the nearest 0.1 kg and to the nearest 0.1 cm, respectively. Waist circumference was measured at the umbilicus level after normal exhalation with participants in standing position. Hip circumference was measured at the maximum circumference of the hips. BMI was calculated as weight in kilograms divided by the square of height in meters (kg/m^2^), and BMI z-score was calculated based on WHO 2007 growth reference using WHO Anthroplus program^20^. Body composition was measured by bioelectrical impedance analysis (BIA) using the InBody 770® (InBody Co., Ltd., Chungcheongnam-do, Korea). This multi-frequency with 8-point tactile electrodes was evaluated in Korean children aged 6–18 years^21^ with 99% reproducibility. Fat mass index (FMI) and fat-free mass index (FFMI) were calculated in the same manner as BMI^22^.

Blood pressure was measured by blood pressure monitor (Dinamap®). The presence of acanthosis nigricans was documented by a pediatrician. Tanner staging was assessed by a self-administered picture questionnaire and verified by a pediatrician.

### Metabolic profiles

Venous blood was obtained after a 12-h fast to evaluate biochemical parameters at the 1st and 6th visits of the study. Fasting plasma glucose (FPG) was measured by the hexokinase method (GLUCOSE, Architech; Abbott Laboratories, Irving, TX). Serum total cholesterol, HDL-C, and triglyceride were measured by enzymatic colorimetric assay (CHOLESTEROL, Architech; ULTRA HDL, Architech; and TRIGLYCERIDE, Architech; Abbott Laboratories). LDL-C was measured by homogeneous liquid selective detergent (DIRECT LDL, Architech; Abbott Laboratories). Serum alanine aminotransferase (ALT) was determined according to the standard of International Federation of Clinical Chemistry (ALANINE AMINOTRANSFERASE, Architech; Abbott Laboratories).

### Statistical analysis

Sample size estimation: With power of 0.80 and alpha level of 0.05, sample size of 55 obese children per group was estimated using SD of 0.267 and minimal clinical difference for BMI z-score change was 0.17.

Baseline characteristics for participants in each group were described in mean and SD or frequency and percentages. One-way ANOVA was used to evaluate the difference in the change of variable outcomes between baseline and the 6th month. Generalized Estimating Equation (GEE) model was used to evaluate the changes in outcomes, which included body weight and adiposity, among three groups over three time points (baseline, three, and six months). To evaluate the effect of time on the difference in outcomes among the three groups, the GEE model with interaction term between time point and group was evaluated using likelihood ratio test. The alpha level of 0.05 was considered statistically significant for all analyses. All analyses were conducted using STATA version 16.1 (STATA Statistical Software: Release 16. College Station, TX: STATA Corp LLC. 2019).

## Results

**Figure 1:**
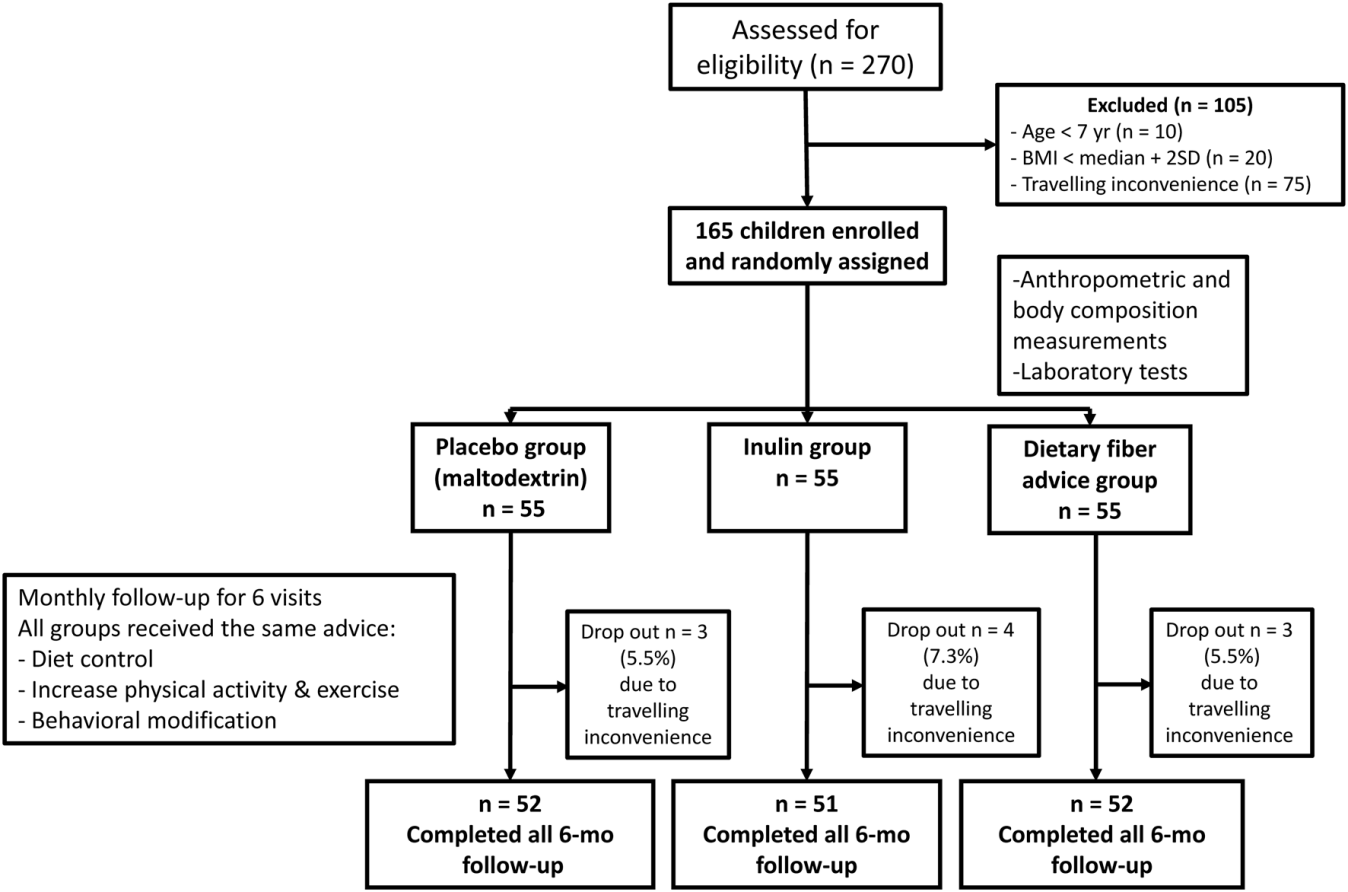
Flow of study participants.

Initially, 270 children were assessed for eligibility through the out-patient clinic (around 10% of the enrollment), and advertisement via social media (around 90% of the enrollment). One-hundred and five children were excluded due to incompatible inclusion criteria. A total of 165 obese children participated in the study (mean age: 10.4 ± 2.2 years, 59% male). They were randomly allocated into the three groups (Fig. 1). Ten participants (6%) dropped out throughout the study due to personal reasons (e.g. travel inconvenience). There was no difference in the attrition rate between the 3 groups.

### Baseline characteristics

Demographic data and baseline characteristics of all groups are illustrated in Table 1. There were no significant differences in the dietary intakes between the three groups; mean daily caloric intake was around 1,400–1,500 kcal. Dietary fiber intake was about 2.8 g/1,000 kcal. Fat and cholesterol intakes were around 57–61 g/day and 300–330 mg/day, respectively. Most participants in the three groups reported balanced caloric distribution intake from the 3-day dietary records. The common features of exercise were brisk walking and cycling in all groups. There were no differences in the amount of time spending for moderate and low intensity exercise. Median screen time in the three groups were similar: around 5.8–7.6 h/day in addition to sedentary activity as shown in Table 1. Overall, the average BMI and BMI z-score were 28 kg/m^2^ and 3.2, respectively. Around four-fifths of the participants reported to have acanthosis nigricans in every group. There were no significant between-group differences in baseline anthropometry, clinical data, and body composition, (P > 0.05).

**Table 1:** Baseline characteristics of obese children at the first visit (n = 165).

	Placebo group (n = 55)	Inulin group (n = 55)	Dietary fiber advice group (n = 55)
Age, years	10.7 ± 2.4	10.3 ± 2.1	10.4 ± 2.0
Male gender, %	56.4	54.6	67.3
**Total nutrient intake**			
Energy intake, kcal/day	1,419 ± 537	1,470 ± 571	1,463 ± 513
Protein intake, g/kg/day	1.53 ± 0.70	1.54 ± 0.57	1.67 ± 0.66
Dietary fiber, g/1,000 kcal	2.8 ± 1.9	2.9 ± 2.3	2.6 ± 1.9
Fat intake, g/day	56.9 ± 28.0	60.9 ± 31.1	58.3 ± 27.4
Cholesterol intake, mg/day	300 ± 198	316 ± 250	330 ± 236
Energy distribution, %C: P: F	48: 16: 36	48: 16: 36	47: 17: 36
**Exercise**			
Low intensity, min/wk^1^	100 (10, 150)	60 (0, 180)	70 (0, 180)
Moderate intensity, min/wk^2^	75 (20, 150)	60 (30, 150)	30 (0, 75)
Sedentary activity, hr/day^3^	4.4 ± 3.0	4.5 ± 3.0	4.6 ± 3.1
BMI, kg/m^2^	28.5 ± 4.6	28.3 ± 4.5	27.4 ± 3.4
BMI for age z-score	3.2 ± 1.1	3.3 ± 1.0	3.2 ± 0.95
WC, cm	90.6 ± 10.8	89.9 ± 11.3	88.6 ± 10.0
SBP, mmHg	115.3 ± 10.0	114.1 ± 8.3	117.4 ± 11.1
Acanthosis nigricans, %	83.6	76.4	80.0
**Tanner stage**			
Stage 1, %	56.4	65.5	69.1
Stage 2, %	10.9	20	12.7
Stage 3, %	20	12.7	10.9
Stage 4, %	10.9	1.8	7.3
Stage 5, %	1.8	0	0
**Body composition (measured by BIA)**			
FMI, kg/m^2^	12.0 ± 2.9	11.9 ± 3.1	11.4 ± 2.7
FFMI, kg/m^2^	16.31 ± 2.61	16.18 ± 1.90	15.98 ± 1.72
Trunk FMI, kg/m^2^	5.7 ± 1.4	5.7 ± 1.5	5.5 ± 1.3
VFA, cm^2^	133.9 ± 39.3	128.8 ± 42.0	125.3 ± 39.7
**Metabolic profiles**			
Total cholesterol, mg/dL	189.8 ± 29.0	189.6 ± 33.7	189.3 ± 32.8
LDL-C, mg/dL	129.5 ± 27.2	130.4 ± 36.7	126.9 ± 30.5
HDL-C, mg/dL	50.1 ± 9.2	50.4 ± 10.2	53.2 ± 9.1
Triglyceride, mg/dL	103.8 ± 52.6	99.3 ± 36.5	101.6 ± 53.2
ALT, U/L	32.8 ± 32.5	31.3 ± 22.8	27.1 ± 17.2
FPG, mg/dL	82.6 ± 5.9	83.7 ± 5.5	83.2 ± 7.4

Data shows means ± SD, median (Q1, Q3) or %. One-way ANOVA was used to evaluate parametric variables, Kruskal–Wallis test was used to evaluate non-parametric variables, and Chi-square was used to evaluate categorical variables. No statistically significant between-group difference was demonstrated for all parameters.

^1^Low intensity (min/wk) was walking from home to school or walking from one place to another for at least 10 min. ^2^Moderate intensity (min/wk) was brisk walking or riding a bicycle continuously for at least 10 min. ^3^Sedentary activity was defined as a type of lifestyle involving little or no physical activity.

ALT, alanine aminotransferase; BIA, bioelectrical impedance analysis; C, cholesterol; FPG, fasting plasma glucose; FFMI, fat-free mass index = fat-free mass (kg)/height (m^2^); FMI, fat mass index = fat mass (kg)/height (m^2^); SBP, systolic blood pressure; VFA, visceral fat area; WC, waist circumference.

Metabolic profiles were not significantly different among the three groups at baseline. The prevalence of high SBP, hypercholesterolemia, high LDL-C, low HDL-C, and hypertriglyceridemia was 7%, 35%, 49%, 11%, and 15% in the placebo group, 3.6%, 38%, 40%, 11%, and 9% in the inulin group, and 16%, 38%, 38%, 7%, and 9% in the fiber advice group, respectively. The prevalence of abnormal FPG in the fiber advice group was 2%, but this prevalence was not observed in the other two groups. These prevalence of abnormal metabolic profiles were not significantly different between the three groups.

### The interventions and confounders

One-hundred and fifty-five children completed the six-month study. The compliance was similar in both groups (number of empty sachets was 85% in the placebo vs. 93% in the inulin group, P = 0.18). Ten percent of participants in inulin group complained about the taste of inulin but they could still consume it until the end of the study. Neither participants who received inulin nor those receiving placebo reported any significant side effects. The compliance in the fiber advice group was determined by the increased amount of fiber after the intervention. Unfortunately, the amount of fiber intake increased similarly in all three groups (from around 2.6 to 6 g/1,000 kcal) without no demonstrable effects of the specific fiber advice. The mean increases ranged from 3.1–3.3 g/1,000 kcal (P < 0.0001) in all groups after the 6-month intervention period.

Obese children in all groups reduced their energy and fat intake as mean reductions ranged from 463–493 kcal/day (P < 0.0001) and 23–30 g/day (P < 0.0001) as per the same standard advice. There was no noticeable difference between groups (Table 2). Physical activity improved with all sorts of intensity and sedentary activity significantly decreased in all groups, except in the placebo group. Nevertheless, there was no between-group difference after the intervention.

**Table 2:** Changes in anthropometry, body composition, and nutrient intake in obese children after receiving inulin, placebo, and dietary fiber advice for 6 months (observed mean of outcomes).

Outcomes	Placebo group (n = 52)			Inulin group (n = 51)			Dietary fiber advice group (n = 52)			Between groups P
	Before	After	Within group P	Before	After	Within group P	Before	After	Within group P	
Energy intake, kcal/day	1,416 ± 542	954 ± 262	< 0.0001	1,447 ± 556	955 ± 242	< 0.0001	1,434 ± 472	968 ± 303	< 0.0001	0.95
Fat intake, g/day	56.6 ± 28.4	33.0 ± 13.1	< 0.0001	59.8 ± 30.0	29.6 ± 11.6	< 0.0001	57.2 ± 27.3	32.3 ± 14.1	< 0.0001	0.47
Dietary fiber, g/1,000 kcal^1^	2.9 ± 1.9	6.0 ± 3.5	< 0.0001	2.8 ± 2.3	5.9 ± 4.4	< 0.0001	2.6 ± 1.9	5.9 ± 4.1	< 0.0001	0.95
**Exercise**										
Low intensity, min/wk^2^	82.5 (32.5, 150)	102.5 (27.5, 217.5)	0.60	80 (30, 150)	105 (40, 210)	0.61	30 (0, 87.5)	130 (37.5, 255)	0.47	0.47
Moderate intensity, min/wk^3^	100 (5, 150)	145 (50, 255)	0.16	70 (5, 200)	100 (50, 150)	0.30	30 (0, 195)	130 (45, 255)	0.0006	0.96
Sedentary activity, hr/day^4^	4.3 ± 3.0	4.6 ± 3.1	0.583	4.6 ± 3.1	3.5 ± 2.3	0.045	4.5 ± 3.0	3.6 ± 1.9	0.045	0.12
BMI for age z-score	3.2 ± 1.1	2.9 ± 1.0	< 0.0001	3.3 ± 1.0	3.0 ± 0.9	< 0.0001	3.2 ± 1.0	2.9 ± 0.9	< 0.0001	0.98
WC, cm	90.6 ± 11.1	89.4 ± 11.7	0.12	89.9 ± 11.6	90.1 ± 11.3	0.86	88.5 ± 10.2	88.7 ± 8.5	0.79	0.33
SBP, mmHg	115.3 ± 10.2	112.4 ± 11.7	0.08	113.6 ± 8.1	112.9 ± 8.6	0.627	118.0 ± 10.7	114.1 ± 9.5	0.008	0.27
**Body composition (measured by BIA)**										
FMI, kg/m^2^	12.0 ± 3.0	11.4 ± 3.2	0.007	12.0 ± 3.2	11.5 ± 3.4	0.006	11.5 ± 2.7	10.6 ± 2.6	< 0.0001	0.25
FFMI, kg/m^2^	16.31 ± 2.61	16.37 ± 2.56	0.38	16.18 ± 1.90	16.38 ± 1.98	0.009	15.98 ± 1.72	16.17 ± 1.55	0.19	0.57
Trunk FMI, kg/m^2^	5.7 ± 1.4	5.5 ± 1.5	0.008	5.8 ± 1.5	5.5 ± 1.7	0.011	5.6 ± 1.4	5.5 ± 1.3	< 0.0001	0.24
VFA, cm^2^	134.1 ± 40.3	128.9 ± 44.1	0.54	130.1 ± 42.9	128.0 ± 46.1	0.38	125.6 ± 40.4	117.3 ± 37.5	0.002	0.11
**Metabolic profiles**										
Total cholesterol, mg/dL	190.2 ± 29.0	185.2 ± 26.8	0.072	191.4 ± 33.1	189.2 ± 37.5	0.47	189.0 ± 33.1	183.7 ± 29.8	0.05	0.69
LDL-C, mg/dL	129.3 ± 27.1	124.5 ± 24.2	0.064	133.0 ± 36.0	133.7 ± 40.2	0.80	126.4 ± 30.7	123.7 ± 28.1	0.30	0.33
HDL-C, mg/dL	50.4 ± 9.4	50.0 ± 9.8	0.69	50.0 ± 9.8	47.6 ± 8.5	0.14	53.3 ± 9.0	52.5 ± 9.5	0.36	0.31
TG, mg/dL	104.1 ± 53.5	115.4 ± 76.8	0.17	99.5 ± 37.5	103.7 ± 49.9	0.51	102.4 ± 54.5	94.5 ± 41.6	0.14	0.13
ALT, U/L	32.0 ± 33.0	23.0 ± 15.8	0.025	31.5 ± 22.1	30.5 ± 40.4	0.79	27.4 ± 17.7	21.3 ± 11.2	0.001	0.21
FPG, mg/dL	82.7 ± 6.0	81.8 ± 5.6	0.29	83.5 ± 5.7	83.7 ± 5.9	0.75	83.4 ± 7.5	83.0 ± 5.7	0.66	0.62

Data was shown as means ± SD and median (Q1, Q3). Pair t-test was used to compare mean difference within group and one-way ANOVA was used to determine differences between groups. Wilcoxon sign test was used to compare non-parametric variables between pre and post intervention within group and Kruskal–Wallis test was used to compare non-parametric changes among three groups.

^1^This amount was from the participants’ diet only and inulin supplementation was not included. ^2^Low intensity (min/wk) was walking from home to school or walking from one place to another for at least 10 min. ^3^Moderate intensity (min/wk) was brisk walking or riding a bicycle continuously for at least 10 min. ^4^Sedentary activity was defined as a type of lifestyle involving little or no physical activity.

ALT, alanine aminotransferase; BIA, bioelectrical impedance analysis; C, cholesterol; FPG, fasting plasma glucose; FFMI, fat-free mass index = fat-free mass (kg)/height (m^2^); FMI, fat mass index = fat mass (kg)/height (m^2^); SBP, systolic blood pressure; VFA, visceral fat area; WC, waist circumference.

### Effects on body weight and adiposity

**Figure 2:**
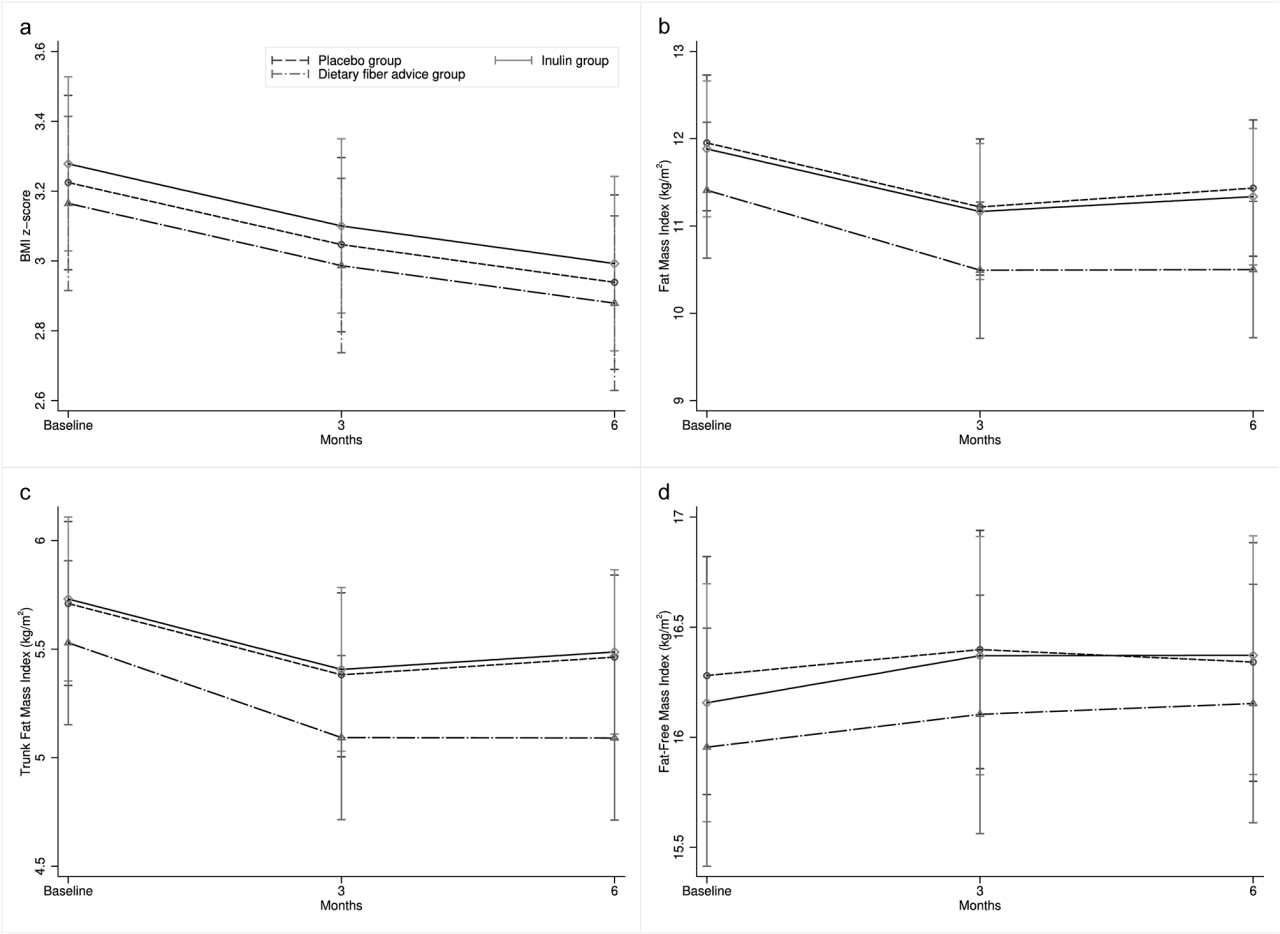
Changes in (**a**) BMI z-score, (**b**) fat mass index, (**c**) trunk fat mass index, and (**d**) fat-free mass index over the 6-month intervention from Generalized Estimating Equation (GEE) model (adjusted mean of outcomes). BMI-z score decreased in all groups (P < 0.0001). Fat mass index (FMI) and trunk FMI rapidly declined in the first 3 months and then remained stable until the 6th month. Fat-free mass index (FFMI) significantly increased in the inulin group through the study (P = 0.009, 95% CI 0.053–0.379), whereas the control and dietary fiber advice groups did not. The GEE slope of FFMI in inulin group increased dramatically, especially in the first 3 months and grew slightly until the 6th month. Between-group comparison shows no significant differences of the changes in BMI z-score, FMI, trunk FMI, and FFMI in 3 time-points (month 0, 3, 6).

All analyses followed the intention-to-treat principle. Within-group comparison showed that BMI-z scores were significantly decreased through the 3- and 6-month interventions in all groups (all significant with P < 0.0001). There was a significant decrease in FMI (placebo: 5%, inulin: 4.2%, fiber: 7.8%) and trunk FMI (placebo: 3.5%, inulin: 5.2%, fiber: 1.8%) in all groups at the 3rd and 6th months compared to baseline (all significant with P < 0.01) (Fig. 2). FMI and trunk FMI rapidly declined in the first 3 months and then remained stable until the 6th month. Difference in the proportion of children in a more advanced tanner stage was not found within the placebo and the inulin groups, but it was observed in the dietary fiber group after the 6-month period. FFMI significantly increased in the inulin group (16.18 ± 1.90 vs. 16.38 ± 1.98 kg/m^2^, P = 0.009) and increased through the study (from GEE model, P = 0.009, 95% CI 0.053–0.379, adjusted for tanner stage, P = 0.033, 95% CI 0.015–0.344) (Fig. 2). In addition, the GEE model showed that the slope of FFMI in inulin group increased dramatically, especially in the first 3 months and grew slightly until the 6th month. VFA significantly decreased in the fiber advice group after the 6-month intervention, but this was not found in the other two groups (Table 2).

Between-group comparisons after the interventions found that there were no significant differences in the changes in BMI z-score, WC, and body composition by ANOVA (Table 2) as well as in the 3 time-points (month 0, 3, 6) during the 6-month intervention period by GEE model (Fig. 2).

### Effects on metabolic outcomes

There were no significant differences within groups in total cholesterol, LDL-C, HDL-C, triglyceride, and FPG after the intervention. There were also no significant differences between groups in metabolic outcomes after the 6-month study period. After the intervention, the prevalence of high SBP, hypercholesterolemia, and high LDL-C decreased in all groups (reduction of 2%, 29%, 35% in the placebo group, 0%, 29%, 35% in the inulin group, and 4%, 27%, 31% in the fiber advice group). On the other hand, the prevalence of low-HDL-C and hypertriglyceridemia remained unchanged after the intervention. The prevalence of abnormal metabolic outcomes was not different within group and among the three groups at the end of the study.

## Discussion

To our best knowledge, this was the largest RCT to evaluate body weight, adiposity, and metabolic outcomes after prebiotic intervention in obese children. We compared the outcomes of inulin supplementation with placebo and dietary fiber advice. After the intervention, BMI z-score and adiposity similarly decreased in all groups, especially in the first 3 months without the demonstrable positive effect of inulin supplementation. However, the significant increase in fat-free mass was observed only in the inulin group during the same period. The overall prevalence of dyslipidemia and clinical signs of insulin resistance was modestly found in all groups. After the intervention, the prevalence was lessened but there was no difference between groups.

### Inulin supplementation

ITF were originally proposed as functional food ingredients capable of improving parameters of the metabolic syndrome^23–25^. Data from previous animal studies have shown that a high-fat diet enriched with ITF led to a decrease in energy intake, less weight gain and adiposity, and a lower serum triglyceride^10^. The researchers showed that glucagon-like peptide (GLP)-1 and GLP-2 contents increased in the proximal colon by ITF fermentation. This suggests that ITF can modulate endogenous production of gut peptides involved in appetite, concentration of ghrelin, and body weight regulation leading to decreased food intake^10^. Our study showed that inulin supplement was well-accepted in children and adolescents, similar to placebo. This finding was in accordance with a previous study in children where ITF was given for 12 weeks^12^. We did not observe any serious side effects from inulin supplementation whereas a previous study by Liber et al. showed that around one-tenth to one-third of children who received ITF of similar dose reported abdominal pain, flatulence, and diarrhea/loose stool^12^. This may be due to different sources and methods of extraction and preparation.

### The effects of inulin supplementation on body weight, body composition, and metabolic outcomes

A recent systematic review and meta-analysis revealed significant reductions in BMI, body weight, and body fat after soluble fiber supplementation in adults with overweight and obesity^11^. The researchers included a total of 12 studies with 609 participants in the meta-analysis. All included studies were placebo controlled RCTs with isolated soluble fiber intervention durations ranging from 2 to 17 weeks. They demonstrated that soluble fiber supplementation significantly reduced BMI by 0.84 kg/m^2^, body weight by 2.52 kg, and body fat by 0.41%, compared with the effects of placebo treatments.

Studies about the impact of ITF in obese pediatric population are still very limited. Liber et al.^12^ studied the effect of ITF supplementation for 12 weeks on the BMI of overweight and obese children. A total of 97 overweight and obese children were randomly assigned to receive placebo (maltodextrin, n = 39) or ITF (oligofructose, n = 40) (age-dependent dose 8–15 g/d). BMI-for-age z-score difference did not differ between the two groups at 12 weeks. The differences between the groups with percentage of body weight reduction and total body fat were not observed, like our study. The researchers concluded that ITF supplementation for 12 weeks had no effect on body weight in overweight and obese children. In this study, the drop-out rates were higher than our study at 19% (at 12 weeks) and 47% (at 24 weeks), probably leading to the indeterminate outcomes. Nevertheless, even with the much lower drop-out rate in our study, we also could not demonstrate the positive outcomes on body weight and adiposity. Considerably, body fat percentage may be not a good indicator of adiposity because high body fat percentage might be due to high adiposity or low lean mass. FMI and FFMI/lean mass index (LMI) can reflect body composition and adiposity better than body fat percentage^26^. Therefore, FMI and FFMI were presented in our study.

Intriguingly, our study showed that inulin supplementation increased FFM despite showing no substantial effect on body weight and adiposity. Even though the effect size was not large, the 1.2% increase in FFMI from baseline was larger than the test-re-test estimates which was around 0.5–1% for Inbody 770. Moreover, the increment remained significant after adjusting for the pubertal stages, which were not change substantially during the 6-month intervention period in the inulin group. So far, there has not been study of inulin on FFM. The plausible mechanism could be described by gut-muscle axis. Inulin might have direct and indirect effects to skeletal muscle by decreasing oxidative stress and inflammation via gut microbiota function. Consequently, muscle glycogen storage is higher and mitochondrial biogenesis and function increase along with the predominance of anabolic signalling pathways, rising the aerobic exercise capacity^27^. Further exploration of this finding may be worthwhile.

There were no significant differences in metabolic profiles between groups from that study^13^ which were the same as our study. At least, we found that the prevalence of high SBP, hypercholesterolemia, and high LDL-C reduced in the participants in all groups after the intervention even though these were not significantly different between groups. The subgroup analysis in the participants with dyslipidemia at baseline also showed no differences among the three groups. These might be due to the proportion of participants who had dyslipidemia at the baseline was limited, so the change of prevalence could not be detected. In addition, it was also noticeable that the effects of the intervention on body weight and composition were most prominent during the first 3 months and then plateau afterwards. Therefore, the difference in the changes in metabolic profiles may have occurred earlier than the 6th month when the second blood test was performed.

### Dose and duration of inulin supplementation

In one double-blind, cross-over RCT studied in 31 non-obese adults, there were no significant differences in body weight after ITF supplementation (10 or 16 g/d for 13 d) compared to the placebo. The researchers reported no differences in appetite sensations and energy intake^28^. The study by Pedersen et al.^29^ determined that the dosage of ITF supplementation may be a crucial factor in appetite regulation. The authors found that ITF supplementation at doses of 35 g/d or higher for 5 weeks in the home environment can suppress hunger. They suggested that ITF raised the concentrations of peptide YY and decreased pancreatic polypeptide concentrations. On the other hand, a RCT involving 97 non-obese adolescents aged 9–13 years reported a significant reduction in body weight (1.3 (SD 0.6) kg, P = 0.048) and BMI z-score difference (0.13 (SD 0.06), P = 0.048) in the ITF group (8 g/d) compared with the control group after 1 year^30^. To recap, the inulin dosage in our study may not be in the range to suppress appetite but in some studies, this dosage has shown to reduce body weight. Nevertheless, it is important to note that the magnitude of difference in BMI z-score in the mentioned study was small at 0.01 SD whereas the mean differences in our study were around 0.3 SD reduction in BMI z-score in all 3 groups which was higher than observed difference in the non-obese population.

### The effects of inulin supplementation vs. behavioral modification

It is known that diet and exercise are important factors affecting body weight and body composition. Although the amount of dietary fiber intake and physical activity in the participants at baseline were very low, based on our intensive behavioral modification and monthly follow-up which was more intense than in normal clinical settings, participants in all groups significantly lessened their energy and fat intake, and improved their dietary fiber intake, exercise as well as sedentary activity. Moreover, they might demonstrate the ‘Hawthorne’ effect. Therefore, we postulated that in our research setting, the effects of inulin supplementation on body weight and adiposity reduction may be overshadowed by the intensive behavioral modification. We showed that the magnitude of the change of BMI z-score and adiposity were the same in all three groups, which were different from the study by Nicolucci et al.^13^ in that their participants did not receive any intensive behavioral modification or frequent follow-up. However, this intensive behavioral modification is very tough to achieve for obese children in real life situation. Therefore, the possibility that inulin supplementation could be beneficial in obesity management without intensive follow-up cannot be ruled out.

### Strengths and limitations of the study

This present study appears to be the largest RCT documenting the change of BMI and body composition after inulin supplementation in obese children. We also extracted inulin from Jerusalem artichoke by using a new technique that has reduced side effects, especially gastrointestinal side effects that occur frequently in participants who receive fructans such as oligofructose^31^ with decent acceptability among children. Another strength of this study was that we did a double-blinded process, except in the dietary fiber group, therefore, we can interpret the results with no bias. Unfortunately, the effects of our intensive behavioral modification and frequent follow-up seemed to be stronger than the additional effects of inulin in reducing BMI and adiposity in obese children. Another limitation of the study was the assessment of exercise and sedentary activity based on parental and participant interview which may not be completely accurate due to item interpretation and recall.

## Conclusions

In conclusion, the intensive behavioral modification and frequent follow-up are effective strategies to reduce BMI and adiposity in obese children. Despite demonstrating no considerable effect on adiposity and metabolic outcomes, inulin supplementation can increase fat-free mass in these children. Further research regarding the change of gut microbiota composition and their metabolites are needed to determine inulin’s impact on host-microbe interaction in obese pediatric population.

## Data Availability

Data described in the manuscript will be made available upon request pending application and approval from the corresponding author.
